# Correction: Wu et al. Homologous Drought-Induced 19 Proteins, PtDi19-2 and PtDi19-7, Enhance Drought Tolerance in Transgenic Plants. *Int. J. Mol. Sci.* 2022, *23*, 3371

**DOI:** 10.3390/ijms232416023

**Published:** 2022-12-16

**Authors:** Caijuan Wu, Miao Lin, Feng Chen, Jun Chen, Shifan Liu, Hanwei Yan, Yan Xiang

**Affiliations:** 1 Laboratory of Modern Biotechnology, School of Forestry and Landscape Architecture, Anhui Agricultural University, Hefei 230061, China; 2 National Engineering Laboratory of Crop Stress Resistance Breeding, College of Life Sciences, Anhui Agricultural University, Hefei 230061, China

The authors wish to make the following corrections to this paper [1]: in the original publication, there was a mistake in Figure 10C,D. The main reason is that we confused the original data in Figure 10B–D when making the bar chart. The corrected Figure 10C,D appears below.

The authors apologize for any inconvenience caused and state that the scientific conclusions are unaffected. This correction was approved by the Academic Editor. The original publication has also been updated.

## Figures and Tables

**Figure 10:**
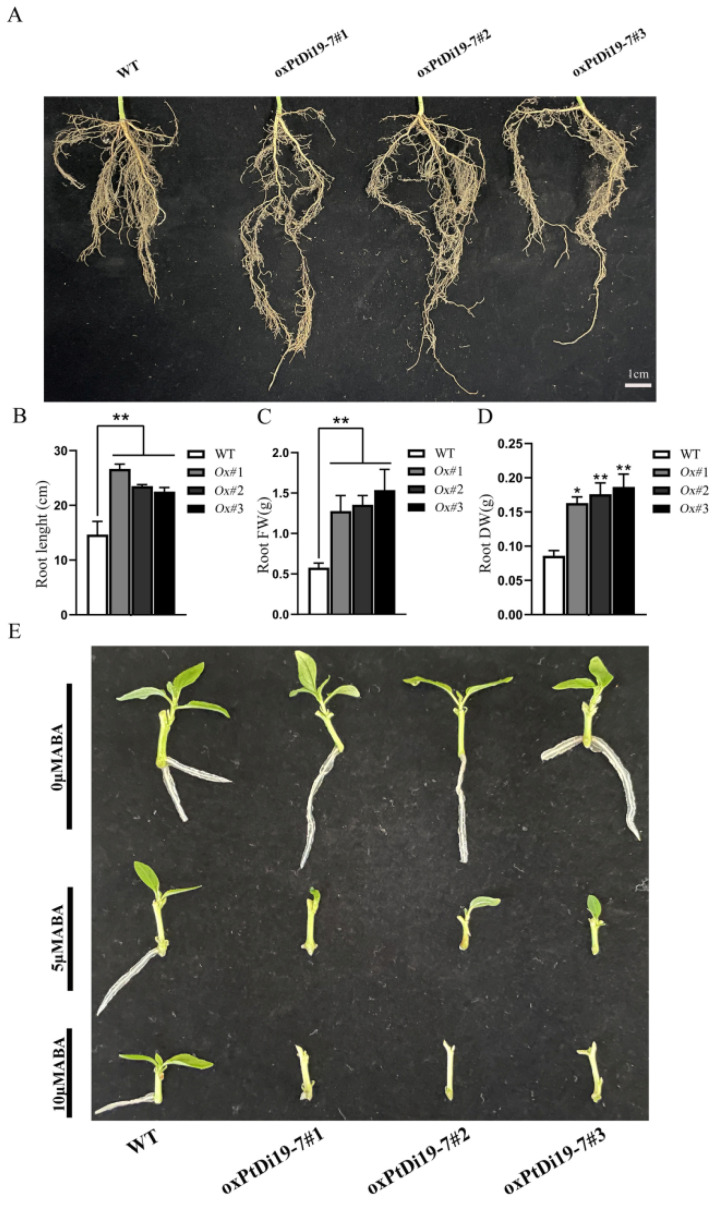
*PtDi19–7* affects root development of transgenic poplar and responds to exogenous ABA. (**A**) Phenotypic analysis of root length after 8 days of drought treatment. (**B**) The root length measurement. (**C**) The root fresh weight. (**D**) The root dry weight. (**E**) Lateral bud outgrowth of short shoot segments grown for 3 weeks on 1/2 MS medium supplemented with ABA (5/10 μM) or without ABA of WT and *oxPtDi19–7* plants. A *p*-value of <0.05 was considered to be significant (*), and a *p*-value of <0.01 was considered to be extremely significant (**).

## References

[1] Wu C., Lin M., Chen F., Chen J., Liu S., Yan H., Xiang Y. (2022). Homologous Drought-Induced 19 Proteins, PtDi19-2 and PtDi19-7, Enhance Drought Tolerance in Transgenic Plants. Int. J. Mol. Sci..

