# Supplementary material for: Homologous Drought-Induced 19 Proteins, PtDi19-2 and PtDi19-7, Enhance Drought Tolerance in Transgenic Plants
Source: Int J Mol Sci. 2022 Mar 21;23(6):3371. doi: 10.3390/ijms23063371 (PMC8954995; doi:10.3390/ijms23063371)
Supplement: Supplementary file 1 [file ijms-23-03371-s001.zip › ijms-1638477-supplementary.pdf]

Supplementary Materials:

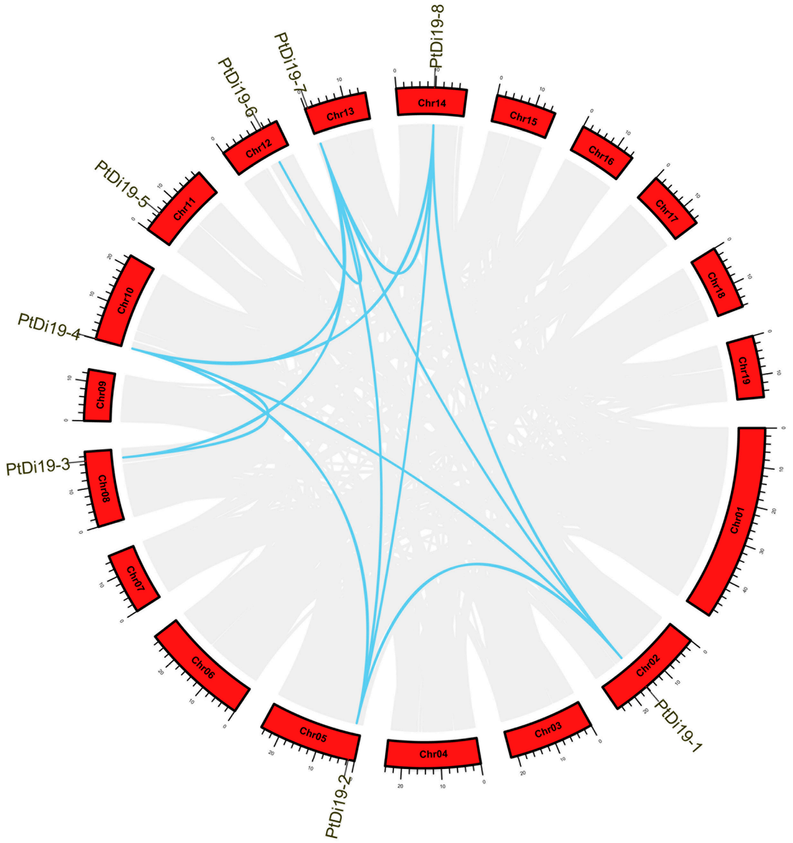

Figure S1. Chromosomal localizations and duplications of Di19 genes on *P. trichocarpa* chromosomes, of which the segmental duplicated Di19 genes indicated with blue lines.

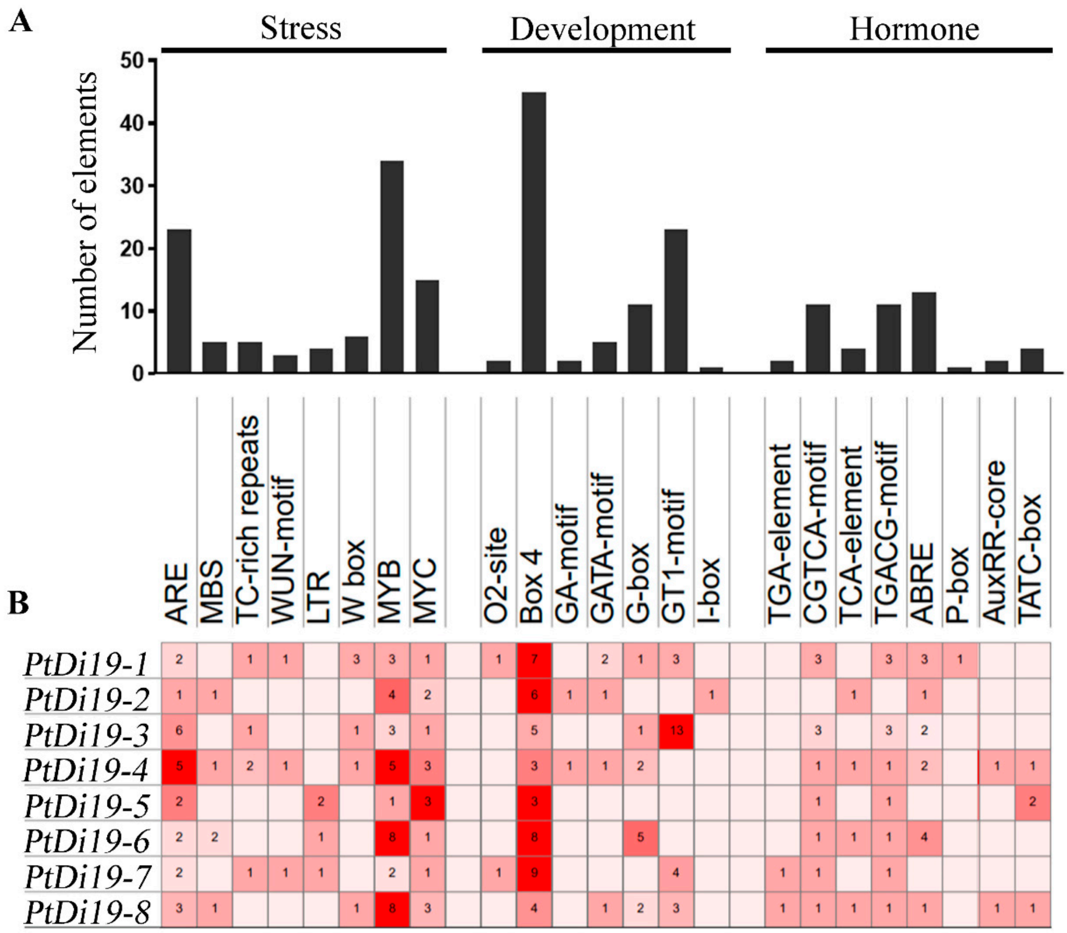

Figure S2. Various cis-acting elements in PtDi19 genes. **(A)** The number of cis-acting elements in response to various factors. **(B)** The number of occurrences of each cis-acting elements.

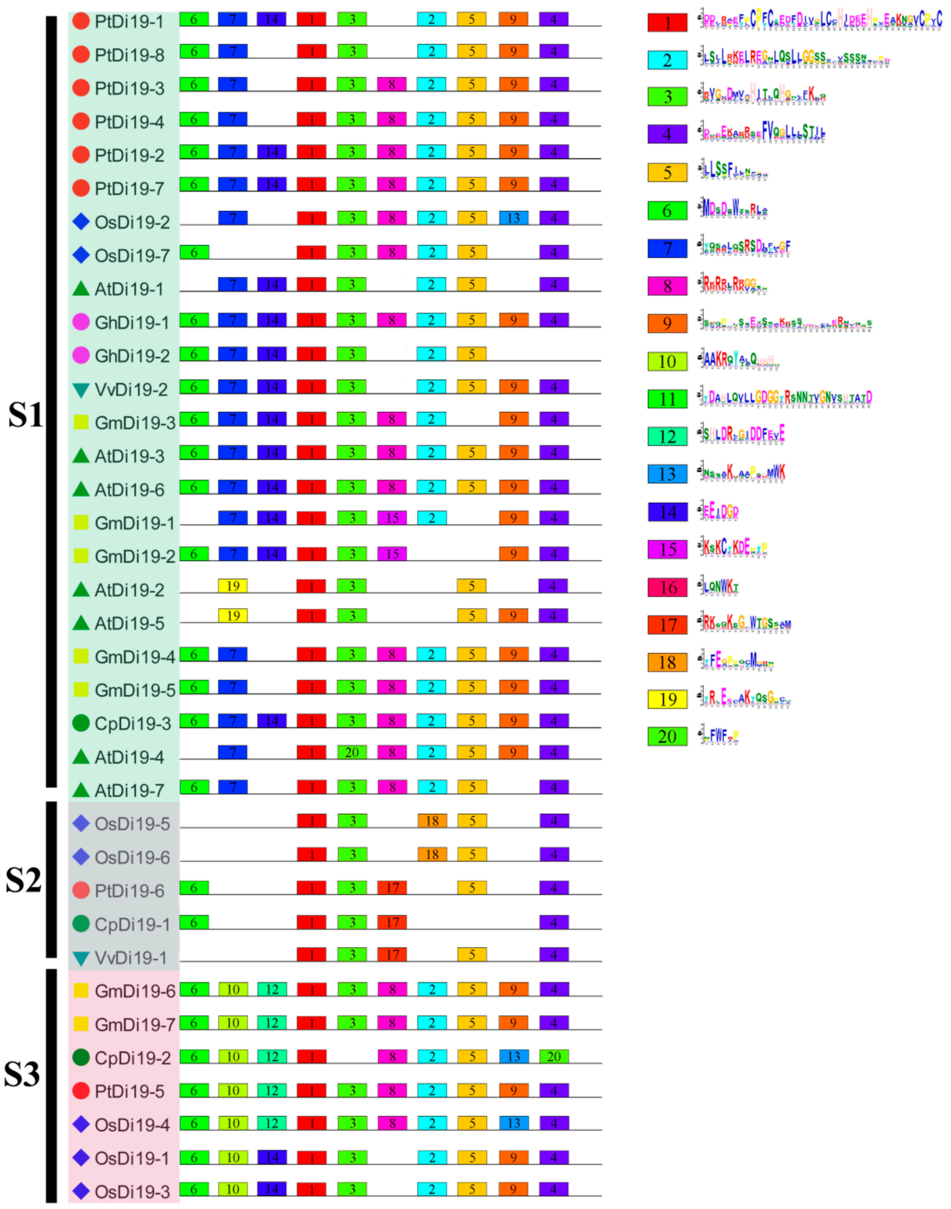

Figure S3. Motif of Di19s in seven species. 20 motifs identified by MEME. Each motif presented with a different-colored box and numbered 1-20 (color Figure online).

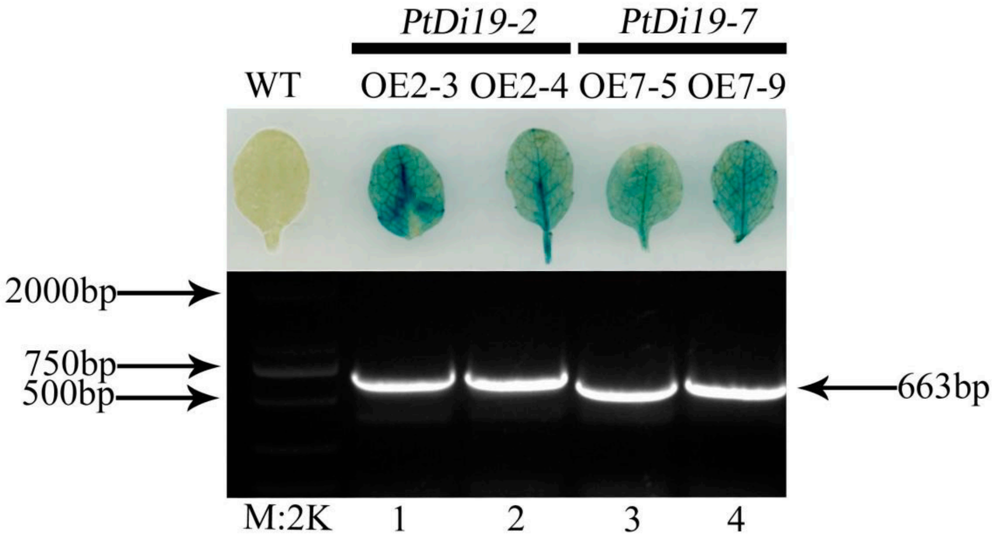

Figure S4. Identification of *PtDi19-2* and *PtDi19-7* transgenic *Arabidopsis.*

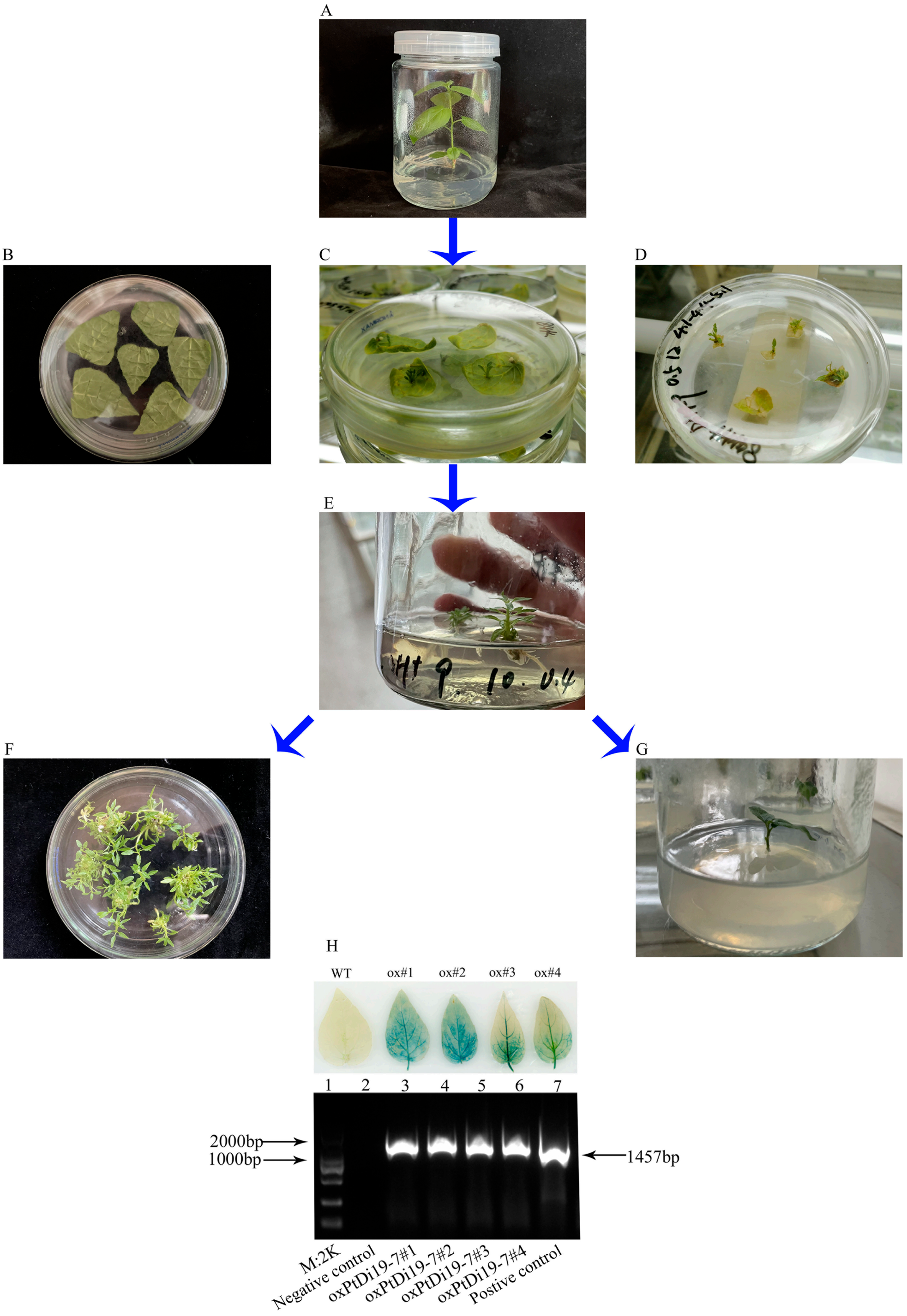

Figure S5. Acquisition and validation of *PtDi19-7* positive plants. **(A)** One-month old tissue culture plantlets of wild-type poplar 84K. **(B)** After infection, 84K poplar leaves were put into co-culture medium for dark culture. **(C)** After three days of dark culture, the leaves were placed into screening differentiation medium. **(D)** The differentiated buds were placed in bud elongation medium. **(E)** The elongated buds were placed in the screening rooting medium. **(F)** Put the leaves into the propagation medium for differentiation. **(G)** The stem segments were inserted into the rooting medium for propagation. **(H)** The four lines were identified by GUS staining and PCR.

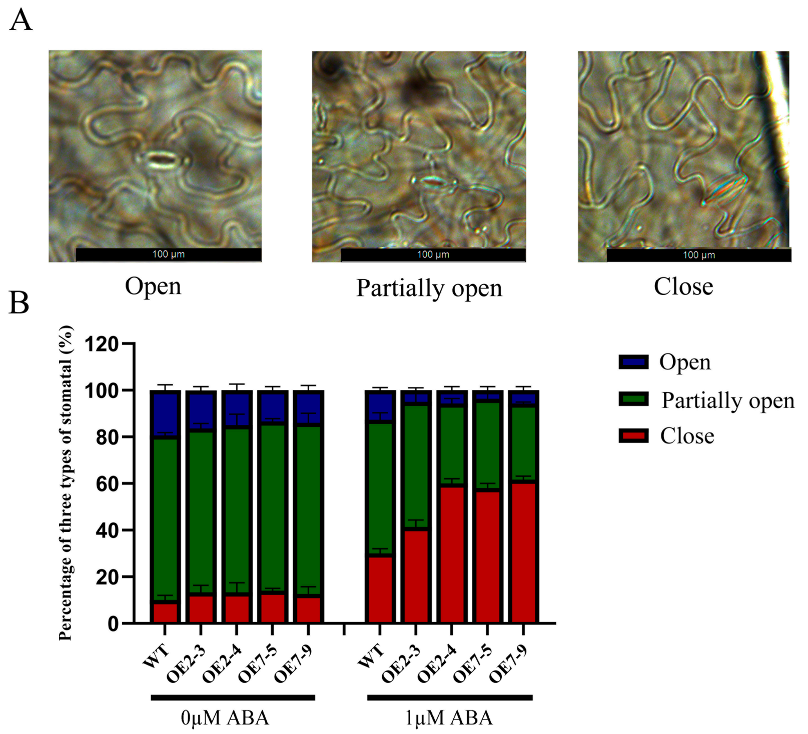

Figure S6. ABA-induced stomatal closure in transgenic *Arabidopsis*. **(A)** Scanning electron microscopy images of three types of stomatal apertures. **(B)** The percentage of three types of stomatal apertures in the leaves of transgenic and WT plants under normal and 1 μM ABA treatment.

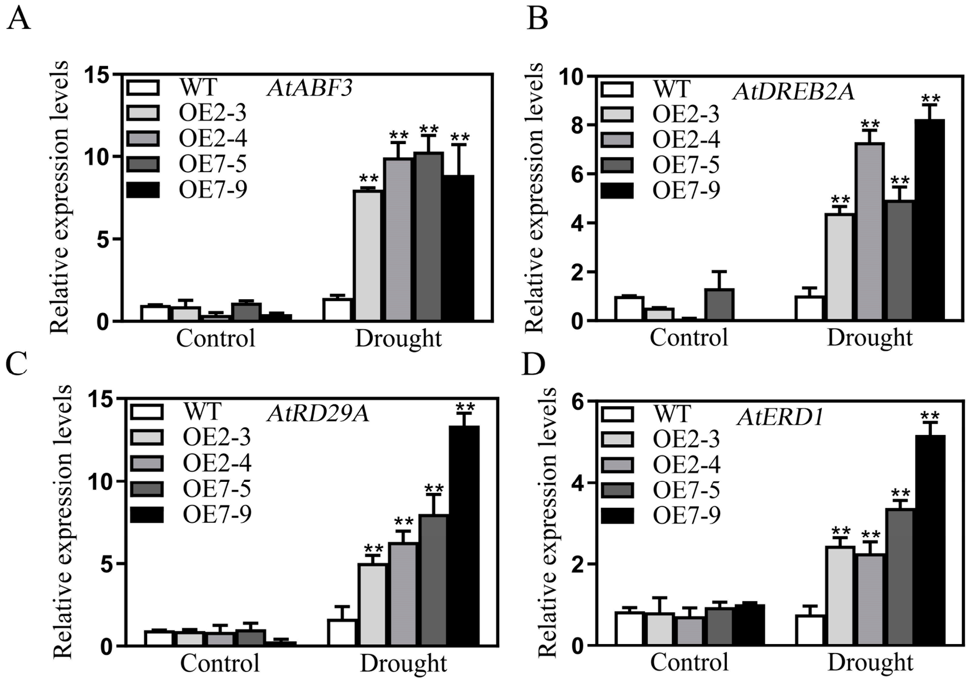

Figure S7. Expression of ABA/drought-related genes of *PtDi19-2* and *PtDi19-7* overexpression in *Arabidopsis*. The transcript levels of *AtABF3* **(A)**, *AtDREB2A* **(B)**, *AtRD29A* **(C)** and *AtERD1* **(D)** measured by qRT-PCR under unstressed conditions or drought treatment for 10 d. ** $p < 0.01$.

Table S1. Drought-induced 19（Di19）identified in *P. trichocarpa* and collected about related physical information.

Name	Gene Identifier	Chr	Location coordinates (5'- 3')	ORF length (bp)	Protein			
					Length (aa.)	PI	Mol.Wt. (Da)	Exons
PtDi19-1	Potri.002G200500	2	16159751--16164658	678	225	4.44	25425.1	5
PtDi19-2	Potri.005G020900	5	1607415--1611963	663	220	5.77	24360.65	5
PtDi19-3	Potri.008G213400	8	16819228--16821768	645	214	5.2	23840.53	5
PtDi19-4	Potri.010G000800	10	98928--101749	648	215	5.23	23974.8	5
PtDi19-5	Potri.011G057200	11	5042374--5046433	702	233	6.17	26389.41	5
PtDi19-6	Potri.012G086500	12	11356226--11359696	627	208	6.23	22994.5	5
PtDi19-7	Potri.013G011200	13	704912--709386	663	220	5.58	24326.49	5
PtDi19-8	Potri.014G125500	14	9660838--9664353	672	223	4.47	25116.84	5

Table S2. Ka/Ks value for duplicate Di19 genes in *P. trichocarpa*.

Pt/Pt	Ka	Ks	Ka/Ks	Duplication date (million years)
PtDi19-1/PtDi19-2	0.347256236	1.65487904	0.209837836	55.16263467
PtDi19-1/PtDi19-7	0.360892376	1.394709067	0.258758177	46.49030223
PtDi19-1/PtDi19-8	0.062087142	0.307389753	0.201981822	10.2463251
PtDi19-1/PtDi19-4	0.393649989	2.249275657	0.175011892	74.97585523
PtDi19-2/PtDi19-7	0.031228281	0.300922206	0.103775264	10.0307402
PtDi19-2/PtDi19-4	0.291104633	1.778188465	0.163708538	59.27294883
PtDi19-3/PtDi19-4	0.050059836	0.202274308	0.247484894	6.742476933
PtDi19-3/PtDi19-7	0.299337727	1.278989763	0.234042317	42.6329921
PtDi19-4/PtDi19-7	0.268509127	1.695637705	0.158352888	56.52125683
PtDi19-6/PtDi19-7	0.689255171	3.088808691	0.223145957	102.9602897
PtDi19-7/PtDi19-8	0.344690151	1.635918177	0.21070134	54.5306059
PtDi19-8/PtDi19-4	0.401384399	1.72237506	0.233041228	57.412502

Table S3. Details of conservative motifs

Motif	Length	Sequence information
1	41	DDLRAEFPCPFCYEDFDIVGLCCHJDEEHPVEAKNGVCPVC
2	32	LSJLRKELREGNLQSLLGGSSCIVSSSNAAPD
3	21	RVGVDMVAHITLQHGNJFKSQ
4	21	DQEEKAKRSEFVQGLLLSTIL
5	11	LLSSFILNFPD
6	11	MDSDSWTSRLA
7	15	YQSALQSRSDLFMGF
8	11	RKRRLRRGGSP
9	29	SKQPSLSSETSSAKKSSDENVLERNVQSS
10	13	AAKRQYALQHHHP
11	29	YDAHLQVLLGDGGYRSNNTVGNVSHTATD
12	14	SHLDRLGIDDFEVE
13	14	NSSAKRAAPSHMWK
14	6	EEIDGD
15	11	KSKCYKDEPYP
16	6	LQNWKT
17	15	RKSQKSGAWTGSSAM
18	12	YFEGPPQCMGRN
19	15	YRLEEAAKYQSGPCQ
20	6	NFWFRP

Table S4. Fluorescence quantitative primer sequence collection

Name	Forward primers	Reverse primers
UBQ10	GTTGATTTTTGCTGGGAAGC	GATCTTGGCCTTCACGTTGT
PtDi19-1	GTGCACAGCCTAGTTTATCAAG	CTGTACGAACTCACTTCTCCTT
PtDi19-2	AATGGGAATGTATCAAACGCAG	GAACCGCAAGTAAAAGTCTCTG
PtDi19-3	CTCGCTTCTCAACTTCTTCAAG	ACCAACAACATCAAAATCCTCG
PtDi19-4	CGTAAGAGAAGATTGCGAAAGG	GAGCTTGCTTCAACAGAAGAAA
PtDi19-5	AGTCTAAAGTCGCCGTTTGTCC	CCCTAACAGCACTTGCAGATGA
PtDi19-6	CAGCATGATGAACCTTTCAACA	GCAAAGCTCTAGAAGATCGTTG
PtDi19-7	GGACAGACTTACTTTCAGGTCA	AATTTCTTGCAGCTAACAAGGC
PtDi19-8	CTTGGCACCTGATCCATTATTG	CCTGTACGAACTCACTTCTCTT

Table S5. Marker genes primer sequences collected

Name	Forward primers	Reverse primers
AtActin	TCGTTGCCCCTCCAGAGA	TACTCTGCCTTTGCGATCCA
AtABF3	CTTTGTTGATGGTGTGAGTGAGC	GTGTTTCCACTATTACCATTGCTG
AtRD29A	TGAAAGGAGGAGGAGGAATGGTTGG	ACAAAACACACATAAACATCCAAAGT
AtDREB2A	GCGATTTTCATCTGGATCACATT	GCAGGTTCCTCGCATCCTT
AtERD1	CCACCGACTCCTCTCTGCTT	AAGGGAGATTCCGAGATATGAAGA

Table S6. Collection of primer sequences related to vector construction

Name	Forward primers	Reverse primers
PtDi19-2	ATGGATGCTGATTCATGGAGTGCT	CTATAAAATATCATCGAGAATTGTAGACAACAACAGCC
PtDi19-7	ATGGATGCTGATTCATGGAGTGCT	CTATAAAATGTCATCAGGAATTGTAGAC
p1301-PtDi19-2	TGCGGTACCATGGATGCTGATTCAT	CGCTCTAGACTATAAAATATCATCGAG
p1301-PtDi19-7	CGGGATCCATGGATGCTGATTCATGG	ACGCGTCGACCTATAAAATGTCATCAGG
p1305-PtDi19-2	TGCTCTAGAATGGATGCTGATTCAT	CGCGGATCCTAAAATATCATCGAGA
p1305-PtDi19-7	GCTCTAGAGCATGGATGCTGATTCA	TCCCCCGGGGGTAAAATGTCATCA
pGBKT7-PtDi19-2	CGGGATCCAATTGTAGACAACAA	CGCGGATCCCTATAAAATATCATCG
pGBKT7-PtDi19-2-N-C	CGGGATCCAATTGTAGACAACAA	CGGGATCCAATTGTAGACAACAA
pGBKT7-PtDi19-2-N	CGGAATTCATGGATGCTGATTCAT	CGGGATCCTCCATGTTGTAGG
pGBKT7-PtDi19-7	GGAATTCCATGGATGCTGATTCA	CGGGATCCCGCTATAAAATGTCA

pGADT7-PtDi19-7	TCCCCCCGGGATGGATGCTGATTCAT	CGGGATCCCTATAAAATGTCATCAGGAATT
